# Postgraduate Medical Education – an increasingly important focus of study and innovation

**DOI:** 10.3205/zma001147

**Published:** 2017-11-15

**Authors:** Martina Kadmon, Olle Ten Cate, Sigrid Harendza, Pascal 0. Berberat

**Affiliations:** 1 University of Augsburg, Medical Faculty, Deanery, Augsburg, Germany; 2 University Medical Center Utrecht, Center for Research and Development of Education, Utrecht, The Netherlands; 3 University Medical Center Hamburg-Eppendorf, III. Department of Internal Medicine, Hamburg, Germany; 4 Technical University of Munich, Medical Education Center, TUM School of Medicine, Munich, Germany

## Editorial

Graduating medical students often feel relieved that the strenuous learning during undergraduate medical education has ended when they eventually hold the license to practice medicine in their hands – just to discover that learning has only just begun when they enter postgraduate training. For many graduates the transition from the final year of undergraduate education to the first year of postgraduate training is rough and demanding with its sudden and unfamiliar burden of professional responsibility. Undergraduate final year medical students participating in a 360-degree realistic full day assessment, simulating a busy first postgraduate working day in hospital [1], [2] reported that patient management and responsibility felt overwhelming at times, even though it was a simulation exercise. They had never truly experienced this merge of required knowledge, skills, and attitudes before. Yet, managing patients and taking up professional responsibilities during postgraduate training are core to every physician’s daily work regardless of specialty.

For many decades, postgraduate medical education in Germany has not been regarded as a training period. Defined learning objectives, structured training programs and validated assessment concepts were missing. Training was based on assumed “learning by doing”, often unsupervised, and completing a set of mostly quantitative requirements, such as numbers of operations, endoscopies, ultrasounds, and other “collectibles”. In other countries, sophisticated admission processes [3], [4] and educational frameworks [5], [6], [7], [8] have been developed for postgraduate medical training programs aiming at preparing residents for their increasingly complex professional roles in delivering health care [9]. The current trend in organizing postgraduate education moves towards competency based training [10] which focuses on individual decisions of entrustment and fading supervision for comprehensive professional tasks depending on the individual learner’s competencies rather than merely counting solitary procedures [11], [12]. To ease the transition from undergraduate to postgraduate training the German National Competence-based Learning Objectives Catalogue (NKLM) has been developed for undergraduate medical education [13]. It is strongly based-on the CanMEDS framework designed for postgraduate medical training and provides a transfer of the framework to undergraduate medical education [13]. Furthermore, the German Medical Association (BÄK) is currently revising the Model Specialty Training Regulations (MWBO) with an emphasis on competences and competence levels [http://www.bundesaerztekammer.de/fileadmin/user_upload/downloads/pdf-Ordner/120.DAET/120DAETFolienBartmann.pdf; accessed: 05.10.2017]. This seems to be a good start towards a more structured postgraduate educational strategy, even though the development is still miles away from the postgraduate educational programs of other countries.

The two most widely accepted frameworks of postgraduate competency based training, the CanMEDS Physician Competency Framework of Canada [9] and the Outcome Project of the Accreditation Council for Graduate Medical Education (ACGME) in the United States [6], follow a system-based approach and operationalise professional activities doctors need to perform in order to provide adequate health care to their patients. Whereas the CanMEDS Framework defines roles of physicians beyond medical expertise focussing on clinicians’ demands in the day-to-day clinical routine, the ACGME Outcome Project specifies core competencies around patient care, system-based practice and their professional development, now with its mandatory biannual reports on the milestones toward competence for every resident [14]. The question that remains is how we can make competencies visible in order to assess them. “A competency is a personal quality, not an action” [12]. It is the basis for professional performance represented by adequate and evidence-based professional activities toward responsible delivery of health care, which essentially is the overriding goal of medical education. The concept of entrustable professional activities (EPAs) may serve as a guide and scaffold for trainers and trainees in designing a postgraduate training curriculum and assessment concept integrating the learner as active participant in his own professional development [15]. An EPA defines a real professional activity, and assessing the trainee’s performance includes all required underlying competencies in an integrated fashion, leading to a stepwise entrustment of a resident with that critical activity, accompanied by a fading level of supervision. This is certainly not an unfamiliar idea. Does it not resemble what we do every day as trainers in postgraduate medical education? And: would it not be helpful to base our entrustment decisions on safer grounds by using common explicit standards?

This special issue on “Postgraduate Medical Education” comprises a large variety of topics including competence-based education, projects on the transition from undergraduate to postgraduate education, different teaching methods, assessment, and career paths in postgraduate medical education. This should especially help to stimulate an evidence-based discourse and consequent development of postgraduate medical education in Germany. Postgraduate educational strategies and structures need to be further developed to provide a framework and scaffold for trainees in a rapidly changing health care system. Trainees need to be accompanied by motivated supervisors who act as role models and provide helpful feedback in an open-minded learning culture. They should be empowered to become actively involved in their postgraduate training and assessment program taking over responsibility for their individual professional development as self-directed learners.

## Competing interests

The authors declare that they have no competing interests. 
